# Temperament and longitudinal changes in physical activity – the Northern Finland Birth Cohort 1966 Study

**DOI:** 10.1186/s12889-023-15303-9

**Published:** 2023-03-03

**Authors:** Anna-Kaisa Karppanen, Jouko Miettunen, Tuula Hurtig, Tanja Nordström, Tuija Tammelin, Raija Korpelainen

**Affiliations:** 1 Research Unit of Population Health, University of Oulu, PO Box 5000, 90014 Oulu, Finland; 2 Department of Sports and Exercise Medicine, Oulu Deaconess Institute Foundation sr, Oulu, Finland; 3 Medical Research Center, Oulu University Hospital and University of Oulu, Oulu, Finland; 4 Research Unit of Clinical Neuroscience, Psychiatry, University of Oulu, Oulu, Finland; 5 PEDEGO Research Unit, Child Psychiatry, University of Oulu, Oulu, Finland; 6 Clinic of Child Psychiatry, Oulu University Hospital, Oulu, Finland; 7 Arctic Biobank, Infrastructure for Population Studies, Faculty of Medicine, Northern Finland Birth Cohorts, University of Oulu, Oulu, Finland; 8 LIKES, JAMK University of Applied Sciences, Jyväskylä, Finland

**Keywords:** Cloninger, Longitudinal, Exercise, Personality

## Abstract

**Background:**

Insufficient physical activity is risk factor for morbidity and premature mortality. This population-based birth cohort study investigated the cross-sectional and longitudinal associations between self-reported temperament at age 31 and self-reported leisure-time moderate to vigorous physical activity (MVPA) levels and changes thereof from the age of 31 to the age of 46 years.

**Methods:**

The study population comprised 3,084 subjects (1,359 male and 1,725 female) from the Northern Finland Birth Cohort 1966. MVPA was self-reported at ages 31 and 46 years. Novelty seeking, harm avoidance, reward dependence, and persistence and their subscales were measured using Cloninger’s Temperament and Character Inventory at age 31. Four temperament clusters were used in the analyses: *persistent*, *overactive*, *dependent*, and *passive*. Logistic regression was used to evaluate the relationship between temperament and MVPA.

**Results:**

The *persistent* and *overactive* temperament profiles at age 31 were positively associated with higher MVPA levels both in young adulthood and in midlife, while the *passive* and *dependent* temperament profiles were associated with lower MVPA levels. The *overactive* temperament profile was associated with a decrease in MVPA levels from young adulthood to midlife among males.

**Conclusion:**

A *passive* temperament profile characterized by high harm avoidance is associated with a higher risk of low MVPA level than other temperament profiles over the life cycle in females. The results suggest that temperament may play a role in determining the level and sustainability of MVPA. Individual targeting and intervention tailoring for promoting physical activity should consider temperament traits.

**Supplementary Information:**

The online version contains supplementary material available at 10.1186/s12889-023-15303-9.

## Background

Insufficient physical activity (PA) is one of the leading risk factors for non-communicable diseases such as cardiovascular diseases, cancer, and diabetes, and has a negative effect on mental health and quality of life. It is also the primary cause of premature death worldwide [1–4]. One-fourth of the world’s population does not reach the minimum recommended levels of PA [5]. Several studies have confirmed the association between moderate to vigorous physical activity (MVPA) and health [6, 7]. Improving PA in general is a public priority, but increasing individuals’ PA levels is challenging. PA is a complex behavior that can change across the lifespan. Researchers have suggested that health behavior counselling should be tailored according to individual characteristics because the same intervention may not be effective for everyone [8]. Identifying the individual characteristics associated with adult PA could help develop tailored interventions for those who need it.

Human behavior is a multifaceted interplay of several components, and one of which is personality. Temperament is a foundation of personality that develops early in life [9] and has been defined as personality components that are inherited [10, 11], emotionally based, and developmentally stable [12]. Temperament has been described in terms of habits and skills that are elicited by simple stimuli perceived by the physical senses, reflects the way in which one approaches and reacts to the world, and influences one’s behavior and the way in which one interacts with others [11]. Understanding one’s temperament can help understand how one reacts and relates to the environment [10].

One of the ways to assess temperament is Temperament and Character Inventory (TCI) which is based on Cloninger’s psychobiological model and divides temperament into four genetically independent temperament traits: novelty seeking, harm avoidance, reward dependence and persistence [13]. Novelty seeking is the tendency to seek excitement, which can manifest itself as imprudent decision-making, rapid loss of temper, extravagance, and active avoidance of frustration [13]. Harm avoidance is characterized by excessive worrying, pessimism, shyness, and being fearful, suspicious, and easily fatigued. Reward dependence is the tendency to respond intensely to reward signals, especially verbal signals of social approval and support. Persistence is the tendency to continue working on something notwithstanding fatigue or frustration [13]. Based on this model, the TCI has been widely used to measure individual differences in these four main temperament traits [13].

Previous studies using the TCI have found that low novelty seeking, and high harm avoidance scores are associated with low MVPA levels and obesity[14] and that exercise dependence is positively associated with high harm avoidance and persistence scores [15]. Several studies have used the five-factor model (FFM) to investigate the association between personality and PA [16, 17]. In line with studies using the TCI, studies using the FFM have shown that higher neuroticism scores are associated with lower PA levels and more sedentary lifestyles, whereas higher extraversion, openness, and conscientiousness scores are associated with higher PA levels [16–18].

To our knowledge, no large population-based longitudinal studies extending to midlife have used information on temperament combined with prospective data of leisure-time MVPA. Most previous studies have used small sample sizes and cross-sectional data which makes it difficult to draw conclusions on the association between temperament and PA level stability. In addition, the growing evidence on temperament indicates that prediction and understanding can sometimes be improved by looking at combinations of temperament attributes rather than temperament traits in isolation [19]. Therefore, we aimed to evaluate cross-sectional and longitudinal associations between temperament and leisure-time MVPA levels from young adulthood (age 31) to midlife (age 46) using Cloninger’s temperament traits and clusters in a large population-based birth cohort. Based on previous literature [14, 16–18], we hypothesized that those with passive temperament profiles especially those who score high in harm avoidance would be physically less active than those with other temperament profiles.

## Methods

### Study design and participants

**Fig. 1:**
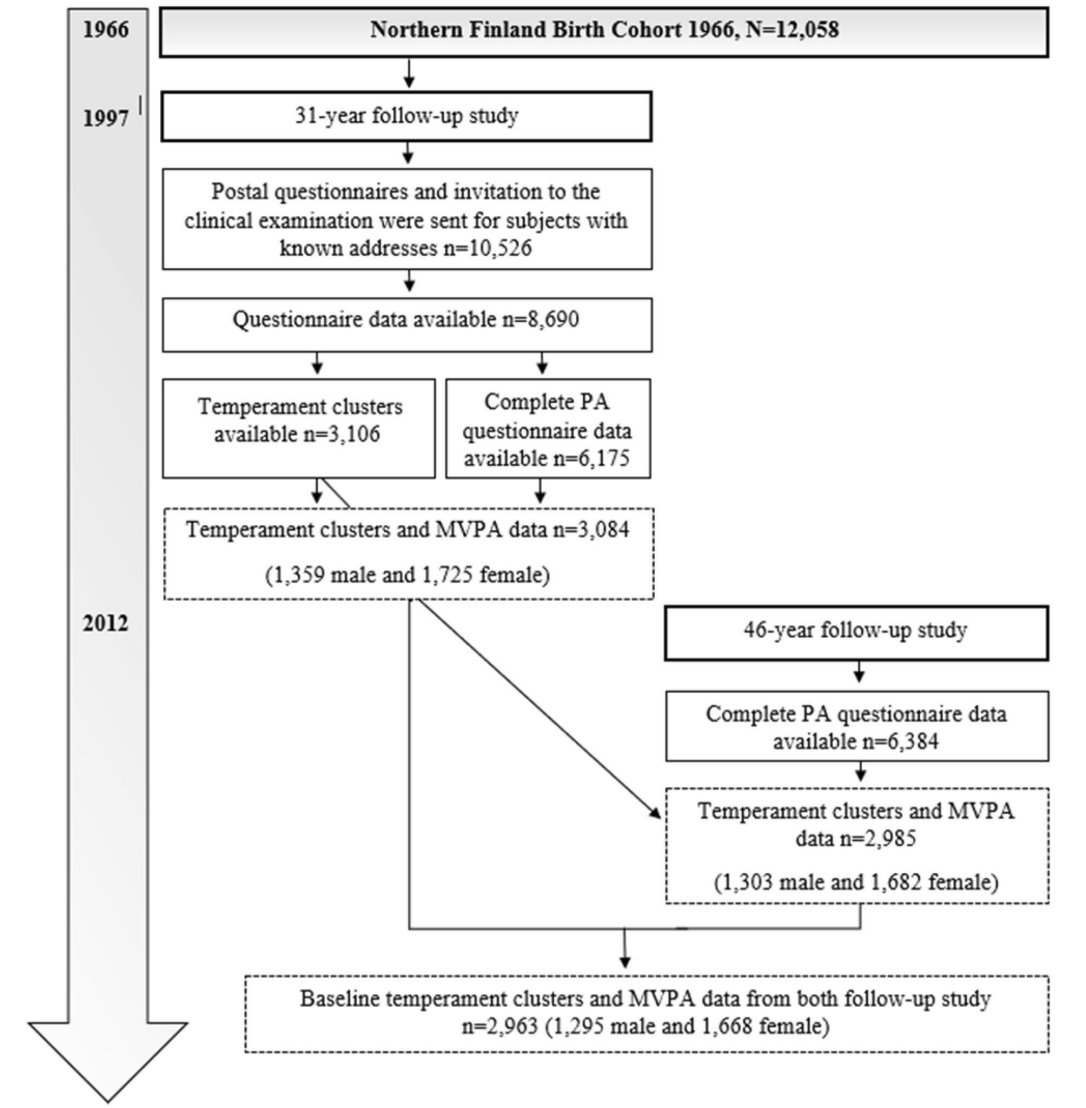
The flow diagram of timeline, content, and participants in the Northern Finland Birth Cohort 1966 study

The study population was based on the Northern Finland Birth Cohort (NFBC) 1966 [20, 21]. The NFBC1966 is an ongoing longitudinal birth cohort of individuals whose expected dates of birth fell in 1966. The cohort members have been carefully monitored from the prenatal period onwards with interviews, postal questionnaires, and clinical health measurements. The subjects and their parents provided written informed consent for the study. Personal identity information was encrypted and replaced with identification codes to ensure full anonymity. This follow-up study was approved by the Ethics Committee of the Northern Ostrobothnia Hospital District, Oulu, Finland (94/2011), and was conducted in accordance with the Declaration of Helsinki. For the cross-sectional analysis, we included data from 3,084 subjects who attended the clinical examination in the 31-year follow-up study [22] and completed MVPA and temperament questionnaires and 2,985 subjects who had completed baseline temperament and MVPA questionnaires in the 46-year follow-up study. For the longitudinal analysis, we included data from 2,963 participants who completed baseline temperament and MVPA questionnaires at ages 31 and 46 years (Fig. 1).

### Measures

#### Temperament

Temperament was measured using Cloninger’s TCI, which includes four genetically independent temperament traits and their subscales: novelty seeking (exploratory excitability, impulsiveness, extravagance, and disorderliness), harm avoidance (worry/pessimism, fear of uncertainty, shyness, and fatigability), reward dependence (sentimentality, attachment, and dependence), and persistence (Cloninger, 1994). The character items of the TCI were not used in the surveys. The TCI (version IX) consist of 240 true/false items, including 107 temperament items (35 harm avoidance, 40 novelty seeking, 24 reward dependence and 8 persistence items). Temperament trait summary scores were calculated for the four main temperament traits.

#### Self-reported leisure-time physical activity

At the ages of 31 and 46 years, the weekly frequency of MVPA was enquired with the following questions: “How often do you participate in MVPA during your leisure-time?”[23]. MVPA was described as inducing at least some sweating and breathlessness. PA frequency had six response options for both intensity levels: (1) once a month or less often, (2) two to three times a month, (3) once a week, (4) two to three times a week, (5) four to six times a week, and (6) daily. The weekly duration of MVPA were enquired with the following questions: How long at a time do you engage in MVPA during leisure time?”[23]. PA duration at a time had the following response options for both intensity levels: (1) not at all, (2) less than 20 minutes, (3) 20–39 minutes (4) 40–59 minutes, (5) 60–90 minutes, and (6) more than 90 minutes [23]. The amount of MVPA were calculated by multiplying its frequency by its duration. The question on the frequency of MVPA has been shown to be associated with physical performance at the age of 31 [24].

The participants were divided into two groups according to weekly MVPA (minutes in a week): (1) low MVPA (0–149 min in a week; not meeting the PA recommendations) and (2) high MVPA (more than 150 min in a week; meeting the PA recommendations) [7]. The participants were then divided into four groups: (1) *stable low* (low MVPA level at the ages of both 31 and 46 years, (2) *increased* (low MVPA level at 31 but high at 46), (3) *decreased* (high MVPA level at 31 but low at 46), and (4) *stable high* (high MVPA level at both 31 and 46).

#### Other variables

The following variables were considered as potential confounders and included as covariates in the analyses of temperament and physical activity: Severity of anxiety and depression at the age of 31, perceived health at the age of 31, the level of education, smoking status, alcohol consumption, marital status, employment status, and perceived health at the age of 46.

The Hopkins symptom checklist (HSCL-25) was used to evaluate the presence and severity of anxiety and depression symptoms during the previous week. It includes 25 items, 10 of which are related to anxiety and 15 to depression [25]. The HSCL-25 score was calculated by dividing the total score (sum score of the items) by the number of items answered and ranged from 1 to 4. The total score was calculated only if no more than five of the 25 values were missing. A cut-off of 1.75 was considered to suggest depressive symptoms [26]. HSCL-25 was further classified as (1) major symptoms and (2) no or minor symptoms.

Education level at the age of 46 years was classified into following categories: (1) No professional education or vocational or college level and (2) University or polytechnic degree.

Smoking status at the age of 46 was classified as (1) current smoker (2) non-smoker and employment status was classified as (1) other and (2) employed. Marital status was classified into following categories: (1) other and (2) Married or cohabiting. Alcohol consumption at the age of 46 was classified as (1) high-risk drinker and (2) abstainer or moderate drinker [27].

At age of 31 and 46 years, perceived health was asked with the question “How would you describe your overall health at the moment with five options (excellent, good, fair, poor, and very poor)?” The options were further categorized into two classes: (1) other (fair, poor, or very poor) and (2) Good (excellent and good) [28].

### Statistical analysis

The characteristics of the study population and physical activity at 31 and 46 years of age were expressed as frequency distributions and means and standard deviations (SD) for normally distributed variables and as medians and interquartile ranges for variables with skewed distributions. The independent *t*-test for continuous variables with normal distribution and the Mann − Whitney *U* test for variables with skewed distribution and Chi-square test for the categorical variables were used to examine differences between the genders. Cross-sectional associations between temperament clusters and the level of MVPA were evaluated using the Kruskal − Wallis test, and the pairwise comparison was made if overall p-value was significant. For the illustrative purpose the means with standard deviation for the MVPA minutes in a week at the age of 31 and 46 years were calculated.

**Fig. 2:**
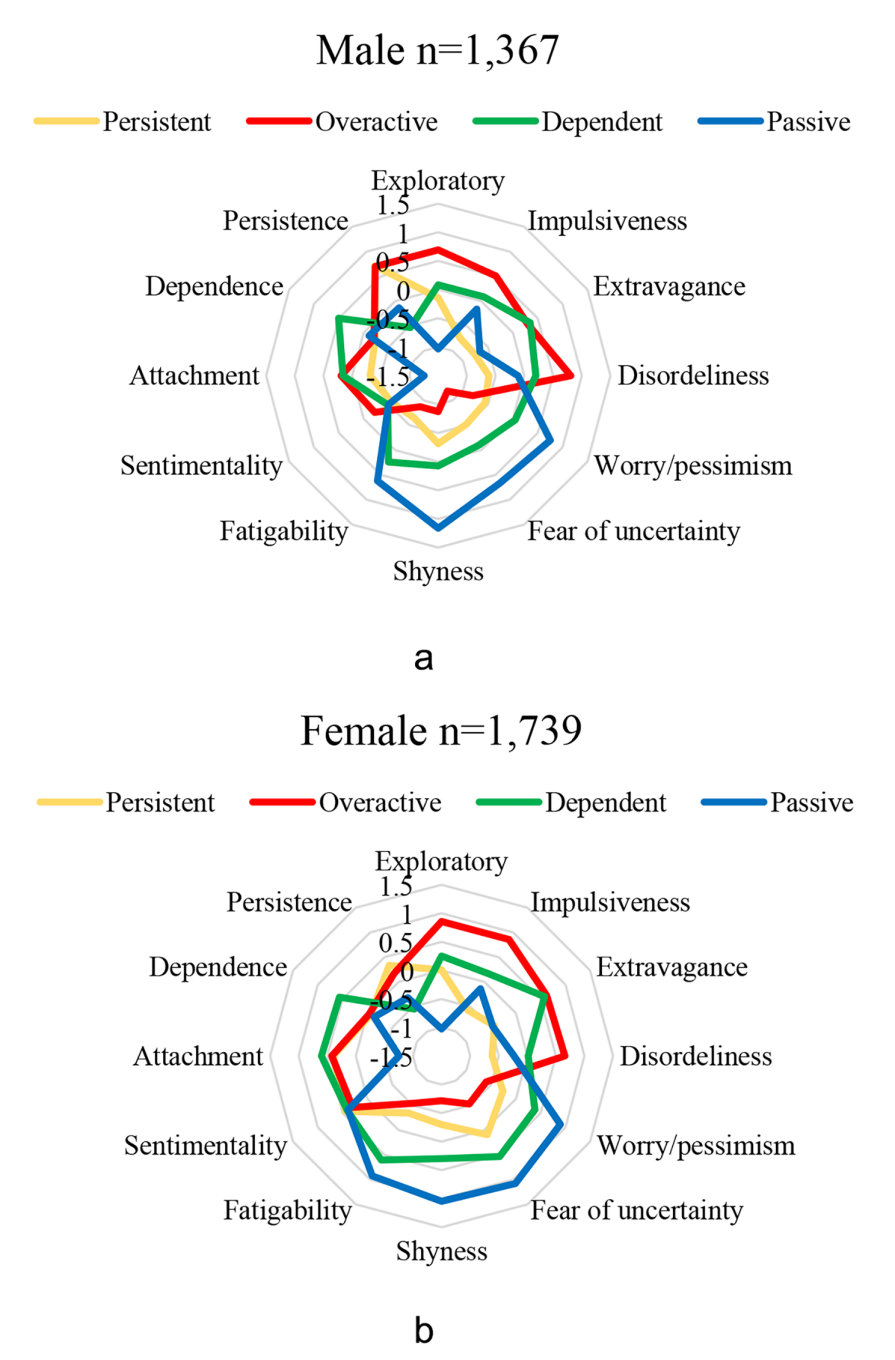
a. Star plots describing the clusters for males. b. Star plots describing the clusters for females

Cluster analysis was performed to form groups of individuals who were as similar to each other as possible while being as different from individuals in other groups as possible in terms of TCI traits. The final cluster model included four distinct clusters according to the temperament trait scores. Cluster 1 individuals were characterized by high persistence (*persistent*), Cluster 2 individuals had high novelty seeking and low harm avoidance scores (*overactive*), Cluster 3 individuals had the highest attachment and dependence scores (*dependent*), and Cluster 4 individuals had high harm avoidance and low novelty seeking scores (*passive*). Clustering was performed separately for males and females. Star plots describing the clusters for males and females are seen in Fig. 2a and b. The average score of each cluster on each of the twelve TCI subscale, with the line closer to the middle of the plot represents lower scores and the line closer to the edge of the plot represents higher scores. The details of the clustering method used in this study have been described previously [29].

To evaluate how temperament clusters associated with longitudinal changes in MVPA levels from young adulthood to midlife, multinominal logistic regression analyses were carried out. The crude and adjusted odds ratios (OR) were used to quantify the strength of the associations between these variables. When predicting longitudinal changes in MVPA levels, stable low was used as a reference. The presence and severity of anxiety and depression symptoms at the age of 31 years and perceived health at the age of 31 years, level of education, smoking status, alcohol consumption, marital status, and perceived health at the age of 46 were treated as covariates in the adjusted models. Covariate was added in the model if it was significantly associated with the independent or dependent variable either in the male´s or female´s model.

Allowing results to be compared to other studies, we also performed the analyses using the scores of the four main TCI traits. At first, associations between TCI scores and MVPA variables were analyzed with Spearman’s rank correlation test. To examine the predictive properties of TCI scores for longitudinal stability and changes of MVPA, TCI scores were transformed to z-scores, and multinominal logistic regression analyses were conducted. Crude and adjusted ORs with 95% confidence intervals (CI) were calculated.

All analyses were conducted separately for males and females since there were differences in physical activity levels and TCI-scores between genders. The level of statistical significance was set to *p* < .05, and all tests were two-tailed. All statistical analyses were performed using IBM SPSS Statistics version 24.

## Results

The characteristics of the study population in midlife and means and standard deviations of temperament traits (TCI scores) across the clusters at the 31 years in males and females are presented in Table 1 and in Additional file 1. In this study, the participants who continued to the 46-year follow-up study and returned PA questionnaires were more often female than male (56% vs. 44%; p < .046). Males who continued to the 46-year follow up study perceived their health more often as very good or good than fair, poor or very poor (66% vs. 45%; p = .040). Females had higher mean (SD) scores than males in novelty seeking (20.7 [6.0] vs. 19.5 [5.9]; p < .001), harm avoidance (15.1 [5.9] vs. 12.8 [6.0]; p < .001), and reward dependence (16.1 [3.4] vs. 13.2 [3.7]; p < .001). Conversely, males scored higher than females in persistence (4.6 [1.8] vs. 4.1 [1.7]; p < .001). Males reported higher MVPA (minutes in a week) than females at both time points (Table 1). Both genders showed a slight increase in MVPA time between the two time points.

**Table 1:** Characteristics of the Northern Finland Birth Cohort (1966) study population at ages 31 and 46 years

Variable	Male	Female	All	*p*-value
Temperament traits at age 31, n (%)	1367 (44)	1739 (56)	3106	
Persistence, mean (SD)	4.6 (1.8)	4.1 (1.7)	4.3 (1.7)	**< .001** ^**a**^
Novelty seeking, mean (SD)	19.5 (5.9)	20.7 (6.0)	20.7 (6.0)	**< .001** ^**a**^
Reward Dependency, mean (SD)	13.2 (3.7)	16.1(3.4)	14.8 (3.9)	**< .001** ^**a**^
Harm avoidance, mean (SD)	12.8 (6.0)	15.1 (5.9)	14.0 (6.1)	**< .001** ^**a**^
MVPA at age 31, n (%)	1359 (44)	1725 (56)	3084	
min/week, md (IQR)	45 (10−150)	45 (15−113)	45 (10−113)	**.034** ^**b**^
min/week, mean (SD)	98.3 (118.4)	82.0 (99.1)	89.2 (108.3)	**<.001** ^**a**^
MVPA at age 46, n (%)	1303 (44)	1682(56)	2985	
min/week, md (IQR)	75 (15−188)	75 (23−188)	75 (15−188)	**.032** ^**b**^
min/week, mean (SD)	107.6 (120.3)	112.6 (119.2)	110.4 (119.7)	**.251** ^**a**^

*Note*. ^a^*p*-value for the difference between male and female (unpaired two-sample *t*-test), ^b^*p*-value for the difference between male and female (the Mann−Whitney *U* Test), Temperament traits – Cloninger’s temperament and Character Inventory, SD – Standard deviation, MD – median, IQR – Interquartile range, MVPA – moderate to vigorous physical activity, min/week – minutes in a week

### Cross-sectional associations between temperament clusters and leisure-time physical activity at ages 31 and 46

At the age of 31 years, TCI and PA questionnaires were returned by 3,084 (1,359 male and 1,725 female) participants. The TCI *persistent*, *overactive*, *dependent*, and *passive* clusters included 28%, 22%, 29%, and 21% of the male participants, and 26%, 24%, 28%, and 22% of the female participants, respectively. Individuals in the *passive* cluster perceived their health more often as fair, poor, or very poor than individuals in other clusters (Male *persistent* 26%, *overactive* 22%, *dependent* 30%, and *passive* 44%, χ^2^[3, N = 1357] = 40.33, p < .001; Female *persistent* 28%, *overactive* 26%, *dependent* 31% and *passive* 43%, χ^2^[3, N = 1721] = 31.14 p < .001). At the age of 46 years, TCI and PA questionnaires were returned by 2,985 (1,303 male and 1,682 female) participants. The TCI *persistent*, *overactive*, *dependent*, and *passive* clusters included 26%, 24%, 28%, and 22% of the male participants, and 26%, 25%, 27%, and 22% of the female participants, respectively.

The self-reported MVPA levels among males and females in the four TCI clusters at the age of 31 and 46 years are presented in Table 2. The MVPA levels differed between the TCI clusters, with the *passive* participants having the lowest PA level in both time points. Post hoc analysis revealed significant difference in MVPA level between the *overactive* and *passive* clusters in both genders in both time points. Post hoc comparisons also showed that the females in the *persistent* and *overactive* clusters reported higher MVPA levels than those in the *dependent* and *passive* clusters.

**Table 2:** Self-reported leisure-time moderate to vigorous physical activity (MVPA) at the age of 31 and 46 years according to TCI clusters at the age of 31 years

	TCI clusters									
Variable	Persistent^1^		Overactive^2^		Dependent^3^		Passive^4^		All	*p*-value^*^
**Male**										
MVPA at age 31, n (%)	377	(28)	304	(22)	390	(29)	288	(21)	1359	
min/week, md (IQR)	45	(10−150)	75	(10−188)	75	(15−113)	38	(0−113) ^b^		**0.008**
min/week, mean (SD)	97	(119)	119	(132)	90	(98)	90	(124)		
MVPA at age 46, n (%)	359	(28)	284	(22)	381	(29)	279	(21)	1303	
min/week, md (IQR)	75	(15−188)	75	(15−188)	75	(15−188)	45	(5−150) ^b^		**0.014**
min/week, mean (SD)	121	(135)	114	(122)	104	(111)	89	(107)		
**Female**										
MVPA at age 31, n (%)	454	(26)	420	(24)	477	(28)	374	(22)	1725	
min/week, md (IQR)	45	(15−113)	75	(15−188)	45	(10−113)^d,e^	30	(0−113)^a,b^		**< 0.001**
min/week, mean (SD)	90	(103)	98	(105)	70	(85)	70	(101)		
MVPA at age 46, n (%)	443	(26)	413	(25)	460	(27)	366	(22)	1682	
min/week, md (IQR)	113	(30−188)	90	(30−188)	75	(15−113)^d,e^	45	(10−113)^a,b^		**< 0.001**
min/week, mean (SD)	134	(131)	126	(127)	95	(112)	94	(119)		

*Note*. ^*^ Overall *p*-value refers to comparison between TCI clusters with the use of independent-Samples Kruskal−Wallis Test. Pairwise comparison was made if overall *p*-value was significant. Adj. p^a-f^ = significance values have been adjusted by the Bonferroni correction for multiple tests

^a^ 1 vs. 4, ^b^ 2 vs. 4, ^c^ 3 vs. 4, ^d^ 1 vs. 3, ^e^ 2 vs. 3, ^f^ 1 vs. 2. Clusters; persistent – characterized by high in persistence, overactive – high in novelty seeking and low in harm avoidance, dependent – highest attachment and dependence, passive – high in harm avoidance and low in novelty seeking, TCI – Cloninger’s temperament and Character Inventory, MVPA– moderate to vigorous physical activity, MD – median, IQR – Interquartile range, SD – standard deviation, min/week – minutes in a week

At the age of 31 years, the rates of belonging to the group of engaging more than 150 min of MVPA in a week were 25%, 34%, 24%, and 22% among males (χ^2^[3, N = 1359] = 12.60, p = .006) and 24%, 28%, 16%, and 15% among females (χ^2^[3, N = 1725] = 29.29, p < .001) in the *persistent*, *overactive*, *dependent*, and *passive* clusters, respectively. At the age of 46 years, the rates of belonging to the group of more than 150 min of MVPA in a week were 32%, 31%, 28%, and 26% among males (χ^2^[3, N = 1303] = 3.73, p = .292) and 37%, 33%, 25%, and 22% among females (χ^2^[3, N = 1682] = 30.04, p < .001) in the *persistent*, *overactive*, *dependent*, and *passive* clusters, respectively.

### Associations between temperament clusters and changes in leisure-time physical activity from age 31 to age 46

The frequencies of TCI clusters and longitudinal changes in MVPA levels from age 31 to age 46 are presented in Table 3. More than half of the participants belonged to the group of engaging less than 150 min of MVPA in a week between young adulthood and midlife.

**Table 3:** Cross-tabulations between temperament clusters and longitudinal changes in weekly moderate to vigorous physical activity (MVPA) levels from age 31 to age 46

	TCI Clusters										
	Persistent		Overactive		Dependent		Passive		All		*p*-value
*Change in MVPA level (min/week) from 31 to 46 years*	n	(%)	n	(%)	n	(%)	n	(%)	n	(%)	
**Male**, n	359		281		378		277		1295		**0.006**
Stable low	200	(56)	141	(50)	220	(58)	176	(64)	737	(57)	
Increased	69	(19)	40	(14)	69	(18)	40	(14)	218	(17)	
Decreased	44	(12)	53	(19)	53	(14)	30	(11)	180	(14)	
Stable high	46	(13)	47	(17)	36	(10)	31	(11)	160	(12)	
**Female**, n	441		407		455		365		1668		**<0.001**
Stable low	231	(52)	225	(55)	295	(65)	252	(69)	1003	(60)	
Increased	107	(24)	72	(18)	88	(19)	58	(16)	325	(19)	
Decreased	47	(11)	48	(12)	47	(10)	35	(10)	177	(11)	
Stable high	56	(13)	62	(15)	25	(5)	20	(5)	163	(10)	

*Note*. *p*-value for Chi-Square Test, TCI – Cloninger’s temperament and Character Inventory, MVPA – moderate to vigorous physical activity, persistent – characterized by high in persistence, overactive – high in novelty seeking and low in harm avoidance, dependent – highest attachment and dependence, passive – high in harm avoidance and low in novelty seeking, Stable low – low MVPA level at ages of both 31 and 46 years, Increased – low MVPA level at 31 but high at 46, Decreased – high MVPA level at 31 but low at 46; Stable high – high MVPA level at ages of both 31 and 46 years, min/week – minutes in a week

Table 4 shows the associations between TCI clusters and longitudinal changes in MVPA levels according to the logistic regression analysis. A stable high MVPA level from young adulthood to midlife was more common among males (OR = 1.76, 95% CI: 1.19 − 2.59, p = .004) and females (OR = 2.12, 95% CI: 1.50 − 3.01, p < .001) in the *overactive* cluster and among females (OR = 1.75, 95% CI: 1.23 − 2.49, p = .002) in the *persistent* cluster. It was less common among females (OR = 0.44, 95% CI: 0.28 − 0.68, p < .001) in the *dependent* cluster and among females (OR = 0.42, 95% CI: 0.26 − 0.68, p < .001) in the *passive* cluster. These associations remained statistically significant after adjusting for presence and severity of anxiety and depressive symptoms at the age of 31 years, perceived health at the age of 31 years, level of education, smoking status, alcohol consumption, employment status, marital status, employment status and perceived health at the age of 46.

**Table 4:** The associations between TCI clusters and longitudinal changes in MVPA levels according to the logistic regression analysis

		TCI clusters								
		Persistent vs. other		Overactive vs. other		Dependent vs. other		Passive vs. other		
		Crude	Adjusted^a^	Crude	Adjusted^a^	Crude	Adjusted^a^	Crude		Adjusted^a^
		OR (95%)	OR (95% CI)	OR (95%)	OR (95% CI)	OR (95% CI)	OR (95% CI)	OR (95% CI)		OR (95% CI)
*Change in MVPA level (min/week) from 31 to 46 years*										
**Male (1295)**	n (%)	359 (28)		281 (22)		378 (29)		277 (21)		
Stable low	737 (57)	1	1	1	1	1	1	1	1	
Increased	218 (17)	1.24 (0.90 to 1.73)	1.11 (0.78 to 1.58)	0.95 (0.64 to 1.40)	0.77 (0.50 to 1.17)	1.09 (0.79 to 1.51)	1.13 (0.80 to 1.60)	0.72 (0.49 to 1.05)	0.98 (0.64 to 1.48)	
Decreased	180 (14)	0.87 (0.61 to 1.27)	0.87 (0.59 to 1.29)	**1.76 (1.22 to 2.55)** ^**^	**1.66 (1.12 to 2.45)** ^*^	0.98 (0.69 to 1.40)	0.89 (0.61 to 1.30)	**0.64 (0.42 to 0.98)**	0.76 (0.49 to 1.20)	
Stable high	160 (12)	1.08 (0.74 to 1.58)	1.01 (0.67 to 1.52)	**1.76 (1.19 to 2.59)** ^**^	**1.64 (1.08 to 2.48)** ^*^	0.68 (0.46 to 1.02)	**0.63 (0.40 to 0.98)** ^*^	0.77 (0.50 to 1.17)	0.97 (0.60 to 1.55)	
**Female (1668)**	n (%)	441 (26)		407 (24)		455 (27)		365 (22)		
Stable low	1003 (60)	1	1	1	1	1	1	1	1	
Increased	325 (19)	**1.64 (1.25 to 1.26)** ^***^	**1.50 (1.12 to 2.03)** ^**^	0.98 (0.73 to 1.33)	1.00 (0.72 to 1.39)	0.89 (0.67 to 1.18)	0.89 (0.66 to 1.21)	**0.65 (0.47 to 0.89)** ^**^	**0.68 (0.48 to 0.97)** ^*^	
Decreased	177 (11)	1.21 (0.84 to 1.74)	1.06 (0.71 to 1.59)	1.29 (0.89 to 1.85)	1.32 (0.89 to 1.95)	0.87 (0.61 to 1.24)	0.83 (0.56 to 1.23)	0.73 (0.49 to 1.09)	0.87 (0.56 to 1.34)	
Stable high	163 (10)	**1.75 (1.23 to 2.49)** ^**^	**1.57 (1.07 to 2.30)** ^*^	**2.12 (1.50 to 3.01)** ^***^	**2.02 (1.38 to 2.95)** ^***^	**0.44 (0.28 to 0.68)** ^***^	**0.42 (0.26 to 0.67)** ^***^	**0.42 (0.26 to 0.68)** ^***^	**0.53 (0.32 to 0.88)** ^*^	

*Note*. ^*^p < .05, ^**^p < .01, ^***^p < .001, reference group: Stable low, TCI – Cloninger’s temperament and Character Inventory, persistent – characterized by high in persistence, overactive – high in novelty seeking and low in harm avoidance, dependent – highest attachment and dependence, passive – high in harm avoidance and low in novelty seeking, OR – Odds Ratio, CI – Confidence intervals, Stable high – high MVPA level at ages of both 31 and 46 years; Increased MVPA – low level at 31, but high at age 46; Decreased – high MVPA level at 31, but low at age 46; Stable low – low MVPA level at ages of both 31 and 46 years. ^a^ adjusted for perceived health at the age of 31, HSCL-25 presence and severity of anxiety and depressive symptoms at the age of 31, level of education, smoking status, alcohol consumption, marital status, and perceived health at the age of 46

A decreasing MVPA level from young adulthood to midlife was more common among males (OR = 1.76, 95% CI: 1.22 − 2.55, p = .003) in the *overactive* cluster. An increasing MVPA level was more common in females (OR = 1.64, 95% CI: 1.25 − 2.26, p < .001) in the *persistent* cluster and less common in *passive* cluster (OR = 0.65, 95% CI: 0.47 − 0.89, p = .007). These associations remained statistically significant after adjusting for presence and severity of anxiety and depressive symptoms at the age of 31 years, perceived health at the age of 31 years, level of education, smoking status, alcohol consumption, employment status, marital status, and perceived health at the age of 46 (Table 4). A decreasing MVPA level from young adulthood to midlife was less common among males in the *passive* cluster (OR = 0.64, 95% CI: 0.42 − 0.98, p = .039), however this associations did not remain significant after the model was adjusted for presence and severity of anxiety and depressive symptoms at the age of 31 years, perceived health at the age of 31 years, level of education, smoking status, alcohol consumption, employment status, marital status, and perceived health at the age of 46.

### Associations between temperament traits and leisure-time physical activity at ages 31 and 46

The correlations between baseline temperament traits (TCI scores) and MVPA levels at the ages of 31 and 46 years are shown in Additional file 2. Lower harm avoidance scores were associated with longer MVPA times in both genders at both time points. Higher reward dependence scores were associated with longer MVPA times among males at the ages of 31 (rho = 0.07; p = .014) and 46 (rho = 0.11; p < .001). Higher persistence scores were associated with longer MVPA times in both genders at the ages of 31 and 46. Higher novelty seeking scores were associated with longer MVPA times at the age of 31 only in females (rho = 0.07; p = .006).

The associations between TCI scores and longitudinal changes in leisure-time MVPA level from the age of 31 to the age of 46 years according to the logistic regression analysis are presented in Additional file 3. Higher novelty seeking scores were associated with decreasing MVPA levels among males (OR = 1.25, 95% CI: 1.06 − 1.47, p = .009) and remained significant after adjusting for presence and severity of anxiety and depression at the age of 31 years, perceived health at the age of 31 years, level of education, smoking status, alcohol consumption, employment status, marital status, and perceived health at the age of 46.

Lower harm avoidance scores were associated with decreasing MVPA levels in both genders and remained after adjusting for above mentioned covariates. Lower harm avoidance scores associated with stability of high MVPA levels in both genders and remained significant after adjusting for covariates in females. Higher reward dependence scores were associated with increasing MVPA levels among males (OR = 1.25, 95% CI: 1.11 − 1.44, p = .007) and remained significant after adjusting for covariates. Higher persistence scores were associated with increasing MVPA levels among females (OR = 1.26, 95% CI: 1.11 − 1.44, p < .001), and with stability of high MVPA levels among males (OR = 1.38, 95% CI: 1.16 − 1.65, p < .001) and females (OR = 1.36, 95% CI: 1.15 − 1.61, p < .001), but also with decreasing MVPA levels among females (OR = 1.27, 95% CI: 1.08 − 1.50, p = .004). The mean TCI scores across the PA groups according to changes in leisure-time MVPA from the age of 31 and to the age of 46 years are presented in Additional file Table 4.

## Discussion

This population-based birth cohort study evaluated the cross-sectional and longitudinal associations between temperament and leisure-time MVPA levels from young adulthood (age 31) to midlife (age 46) using Cloninger’s temperament traits and clusters and a large data set from the NFBC1966. The results suggest that temperament may play a role in determining the level and sustainability of MVPA.

As expected, those with a *passive* temperament profile (high in harm avoidance and low in novelty seeking) reported lower levels of leisure-time MVPA at both time points, especially when compared to those with an overactive profile (high in novelty seeking and low in harm avoidance). Stability of high MVPA levels were less common among males and females with a *dependent* temperament profile (highest attachment and dependence) and among females with a *passive* temperament profile. Moreover, as expected, lower harm avoidance scores were associated with longer MVPA times. Stability of high MVPA level from young adulthood to midlife were more common among males and females with an *overactive* temperament profile (high in novelty seeking and low in harm avoidance) and among females with a *persistent* temperament profile. However, decreasing MVPA levels were associated with an overactive temperament profile (high novelty seeking, low harm avoidance) among males.

Several studies have investigated the association between PA and personality as measured by the FFM [17, 18]. Cloninger’s psychobiological model has been shown to have important similarities to the FFM [30]: Harm avoidance has been found to be positively related to neuroticism; novelty seeking has been shown to be positively related to extraversion; persistence has been shown to be positively related to conscientiousness; and reward dependence has been shown to be positively related to agreeableness and extraversion [31, 32].

Our findings are consistent with previous studies suggesting that higher harm avoidance scores are associated with lower MVPA levels [14] and overall PA levels [17, 18, 33]. High harm avoidance has also been associated with anxiety [34], depression [35], pain responsiveness [36] and weight gain [37]. As a tendency to feel negative emotions, such as worriedness, insecurity, and shyness, in social situations [38], harm avoidance may inhibit the willingness to cope with unfamiliar situations and reduce the exposure to opportunities to be physically active [17, 39]. Approaches that consider past PA experiences and address individual barriers to PA might improve PA counselling outcomes.

Reward dependence is characterized by social attachment. In our study, higher reward dependence scores were associated with increasing MVPA levels among males but also with low MVPA levels at young adulthood and midlife in both genders. A previous study reported that individuals with a *dependent* temperament profile (highest attachment and dependence) scored low on scales for psychosis proneness scales as well as on the hypomania personality scale, which is likely to reflect low energy levels [29]. It has been suggested that individuals who score high in reward dependence may benefit from group interventions, as they may be especially suited to using social support and to be motivated by social norms and group pressure [37].

Our findings are in line with a previous study indicating that a *conscientiousness* temperament (the tendency to be organized and disciplined), which is conceptually like the *persistence* temperament trait of the TCI, was positively associated with higher MVPA levels [18]. It has been suggested that for individuals who score high in such temperament trait, the motivation for PA tends to have internal rather than external sources [40]. Persistent people tend to be industrious and hard-working despite fatigue and frustration, and they intensify their efforts in response to anticipated rewards [13]. In our study, individuals with a *persistent* profile had the highest PA questionnaire response rates.

Our results are also in line with previous findings suggesting that extraversion and sensation seeking, traits similar to novelty seeking, are associated with higher vigorous PA levels [33, 41]. In our study, individuals with an *overactive* temperament profile (high in novelty seeking and low in harm avoidance) had higher MVPA levels than individuals with a *passive* temperament profile at the ages of both 31 and 46 years. However, a decrease in MVPA from young adulthood to midlife was associated with novelty seeking at age 31 among males. The link between novelty seeking and MVPA may be due to a heightened tendency to seek strong sensation stimuli and excitement, which might be fulfilled through MVPA [11, 17] and participation in high-risk sports [42]. Individuals who score higher than average in novelty seeking and average in the other traits have been characterized as impulsive, excitable, extravagant, and disorderly. They are readily engaged in new interests and activities, but they tend to neglect details and are quickly distracted and bored. They are also easily provoked to prepare for flight or fight [43]. It has also been reported that higher novelty seeking is associated with several unhealthy lifestyles and risky behaviors such as smoking [44], eating disorders [45], alcohol use [44, 46], or even drug abuse [47]. Nevertheless, a previous study has reported that novelty seeking decreases with age [48]. Therefore, the fall in novelty seeking can also appear as a decrease in leisure-time MVPA.

The main strengths of this study are its population-based prospective birth cohort design with a large sample size, relatively high response rates, and a long follow-up period. The participants were born in the same geographic area and were of the same age. Certain limitations should also be acknowledged. The temperament identification was based on self-administered questionnaires and no single questionnaire captures all aspects of temperament. Moreover, our results are limited to TCI, which has, however, been widely used to measure individual differences in these four main temperament traits. TCI clusters are based on the NFBC 1966 data. However, advantages of using temperament clusters are that in many cases the clusters are more strongly associated with dependent variables than the individual temperament traits and clusters comprised of multiple temperament traits capture more information about individual differences and risk profile [29, 49]. Yet allows for a more simpler data structure and less statistical tests [29].

It is noteworthy that temperament may influence the discrepancy between self-reported and accelerometer-measured PA. A previous study reported that extraversion, trait similar to novelty seeking, was associated with reporting higher PA level compared to accelerometer-measured PA in college students [17]. It has also been reported that females with an *overactive* profile (high in novelty seeking and low in harm avoidance) may tend to underestimate their weight in postal questionnaires compared to measurements at the physician’s office [29]. This suggests that caution should be exercised when interpreting self-reported positive lifestyles and health-related variables [29]. Finally, the information on the frequency and intensity of PA at the age of 31 and 46 years were based on self-administered questionnaires and thus susceptible to response bias [50]. Measuring physical activity with an accelerometer would have strengthened the validity of the study [51]. Unfortunately, we did not have accelerometer measurement available at the age of 31 years. Although PA questionnaires tend to overestimate the true PA levels, they can provide essential information about PA behavior or perceived time spent in specific activities in adults and are used for grouping people into categories based on their physical activity [39, 52].

## Conclusion

This population-based birth cohort study provides new evidence on the relationship between temperament and longitudinal changes in leisure-time MVPA from young adulthood to middle age. Stable high MVPA level were less common among males and females with a *dependent* temperament profile (highest attachment and dependence) and among females with a *passive* temperament profile (high harm avoidance and low novelty seeking). Stable high MVPA level from young adulthood to midlife were more common among males and females with an *overactive* temperament profile (high in novelty seeking and low in harm avoidance) and among females with a *persistent* temperament profile. Identifying individuals with insufficient PA may help device personalized interventions for promoting PA at the individual and population levels. Individuals high in novelty seeking could benefit from personalized life and PA counselling taking into account their temperament, as well as from feedback strategies to help change their behavior. Information about temperament can also guide health care professionals to identify individual barriers and enablers of PA and personalize behavior counselling. Future therapeutic and PA approaches aiming to prevent physically inactive lifestyles should also consider temperament traits.

## Electronic supplementary material

Below is the link to the electronic supplementary material.

Supplementary Material 1

Supplementary Material 2

Supplementary Material 3

Supplementary Material 4

## Data Availability

The NFBC data is available from the University of Oulu, Infrastructure for Population Studies. Permission to use the data can be requested for research purposes via an electronic material request portal. In the use of the data, we follow the EU general data protection regulation (679/2016) and the Finnish Data Protection Act. The use of personal data is based on cohort participants’ written informed consent at their latest follow-up study, which may cause data use limitations. Please contact the NFBC project center (nfbcprojectcenter@oulu.fi) and visit the cohort website (www.oulu.fi/nfbc) for more information.
