# Supplementary material for: Temperament and longitudinal changes in physical activity – the Northern Finland Birth Cohort 1966 Study
Source: BMC Public Health. 2023 Mar 3;23:426. doi: 10.1186/s12889-023-15303-9 (PMC9985204; doi:10.1186/s12889-023-15303-9)
Supplement: Supplementary file 2 — Supplementary Material 2 [file 12889_2023_15303_MOESM2_ESM.docx]

**Additional file 2**

Spearman correlations between baseline temperament traits (TCI score) and leisure-time moderate to vigorous

physical activity (MVPA) at the ages 31 (1,359 male and 1,725 female) and 46 (1,303 male and 1,682 female) years.

|  | 31 years | |  |  | 46 years | |  |
| --- | --- | --- | --- | --- | --- | --- | --- |
|  | MVPA min/week | |  |  | MVPA min/week | |  |
|  | rho | *p*-value |  |  | rho | *p*-value |  |
| *Temperament traits at the age of 31 years* |  |  |  |  |  |  |  |
| **Male** |  |  |  |  |  |  |  |
| Novelty seeking | .036 | .181 |  |  | .001 | .964 |  |
| Harm avoidance | - .099 | **< .001** |  |  | - .108 | **< .001** |  |
| Reward dependency | .067 | **.014** |  |  | .109 | **< .001** |  |
| Persistence | .054 | **.045** |  |  | .113 | **< .001** |  |
| **Female** |  |  |  |  |  |  |  |
| Novelty seeking | .066 | **.006** |  |  | - .020 | .423 |  |
| Harm avoidance | - .157 | **< .001** |  |  | - .124 | **< .001** |  |
| Reward dependency | .035 | .143 |  |  | .012 | .622 |  |
| Persistence | .110 | **< .001** |  |  | .116 | **< .001** |  |

*Note.* rho – Spearman correlation, TCI – Cloninger’s temperament and Character Inventory,

MVPA – moderate to vigorous physical activity, min/week – minutes in a week
