# Supplementary material for: Temperament and longitudinal changes in physical activity – the Northern Finland Birth Cohort 1966 Study
Source: BMC Public Health. 2023 Mar 3;23:426. doi: 10.1186/s12889-023-15303-9 (PMC9985204; doi:10.1186/s12889-023-15303-9)
Supplement: Supplementary file 3 — Supplementary Material 3 [file 12889_2023_15303_MOESM3_ESM.docx]

**Additional file 3**

Association between baseline temperament traits (TCI score) and change in leisure-time moderate to vigorous physical activity (MVPA) from age 31 to age 46 years according to the logistic regression analysis.

|  |  |  |  |  |  |  |  |
| --- | --- | --- | --- | --- | --- | --- | --- |
|  |  | Increased |  | Decreased |  | Stable high |  |
|  | Stable low | Crude  OR (95% CI) | Adjusted^a^  OR (95% CI) | Crude  OR (95% CI) | Adjusted^a^  OR (95% CI) | Crude  OR (95% CI) | Adjusted^a^  OR (95% CI) |
| *Temperament traits at the age of 31* |  |  |  |  |  |  |  |
| **Male, n (%)** | 737 (57) | 218 (17) |  | 180 (14) |  | 160 (12) |  |
| Novelty seeking | Ref. | 1.00 (0.86 to 1.16) | 1.00 (0.85 to 1.18) | **1.25 (1.06 to 1.47)^**^** | **1.23 (1.03 to 1.47)^*^** | 1.02 (0.86 to 1.22) | 1.08 (0.89 to 1.30) |
| Harm avoidance | Ref. | 0.87 (0.74 to 1.01) | 1.06 (0.89 to 1.26) | **0.74 (0.63 to 0.88)^**^** | **0.77 (0.64 to 0.93)^**^** | **0.76 (0.64 to 0.91)^*^** | 0.83 (0.68 to 1.01) |
| Reward dependency | Ref. | **1.25 (1.06 to 1.46)^**^** | **1.22 (1.03 to 1.46)^*^** | 1.01 (0.85 to 1.19) | 0.96 (0.80 to 1.15) | 1.19 (0.99 to 1.43) | 1.15 (0.94 to 1.40) |
| Persistence | Ref. | 1.16 (1.00 to 1.35) | 1.12 (0.95 to 1.32) | 1.13 (0.96 to 1.33) | 1.14 (0.96 to 1.35) | **1.38 (1.16 to 1.65)^***^** | **1.37 (1.14 to 1.66)^**^** |
| **Female, n (%)** | 1003 (60) | 325 (19) |  | 177 (11) |  | 163 (10) |  |
| Novelty seeking | Ref. | 0.95 (0.84 to 1.08) | 0.99 (0.87 to 1.14) | 1.15 (0.98 to 1.35) | 1.13 (0.95 to 1.35) | 1.07 (0.91 to 1.27) | 1.11 (0.93 to 1.32) |
| Harm avoidance | Ref. | **0.84 (0.74 to 0.96)^**^** | 0.88 (0.76 to 1.03) | **0.78 (0.66 to 0.92)^**^** | **0.81 (0.67 to 0.98)^*^** | **0.58 (0.49 to 0.70)^***^** | **0.62 (0.51 to 0.76)^***^** |
| Reward dependency | Ref. | 1.00 (0.87 to 1.15) | 0.97 (0.83 to 1.13) | 0.96 (0.80 to 1.15) | 0.90 (0.74 to 1.10) | 1.08 (0.89 to 1.31) | 0.99 (0.81 to 1.21) |
| Persistence | Ref. | **1.26 (1.11 to 1.44)^***^** | **1.31 (1.14 to 1.51)^***^** | **1.27 (1.08 to 1.50)^**^** | **1.25 (1.04 to 1.50)^*^** | **1.36 (1.15 to 1.61)^***^** | **1.43 (1.19 to 1.72)^***^** |

*Note*. ^*^*p* < .05, ^**^*p* < .01, ^***^*p* < .001, reference group: Stable low, OR – Odds Ratio, CI – Confidence intervals, TCI score – Cloninger’s Temperament and Character Inventory z-scores, Stable high – high MVPA level at ages of both 31 and 46 years; Increased – low MVPA level at 31, but high at age 46; Decreased – high MVPA level at 31, but low at age 46; Stable low – low MVPA level at ages of both 31 and 46 years. ^a^ adjusted for perceived health at age 31 and HSCL-25 presence and severity of anxiety and depressive symptoms at age 31, level of education, smoking status, alcohol consumption, marital status, and perceived health at age 46.
