# Supplementary material for: Temperament and longitudinal changes in physical activity – the Northern Finland Birth Cohort 1966 Study
Source: BMC Public Health. 2023 Mar 3;23:426. doi: 10.1186/s12889-023-15303-9 (PMC9985204; doi:10.1186/s12889-023-15303-9)
Supplement: Supplementary file 4 [file 12889_2023_15303_MOESM1_ESM.odt]

Additional file 1 

Characteristics of the Northern Finland Birth Cohort 1966 study population at 46 years.

|  | TCI clusters |  |  |  |  |
| --- | --- | --- | --- | --- | --- |
| Variable | Persistent | Overactive | Dependent | Passive | p-value |
| Male |  |  |  |  |  |  |  |  |  |
| Age, years, mean (SD) | 46.6 | (0.6) | 46.6 | (0.6) | 46.6 | (0.6) | 46.5 | (0.6) | .465 |
| Height, (cm), mean (SD) | 178.2 | (6.1) | 178.5 | (6.1) | 178.5 | (5.8) | 177.4 | (6.5) | .117 |
| Weight, (kg), mean (SD) | 85.9 | (14.0) | 87.3 | (14.1) | 86.7 | (15.1) | 84.0 | (13.0) | .046 |
| BMI, (kg/m2), mean (SD) | 27.0 | (4.2) | 27.4 | (3.9) | 27.2 | (4.3) | 26.7 | (4.0) | .345 |
| WC, (cm), mean (SD) | 96.1 | (11.4) | 97.7 | (10.7) | 97.5 | (12.1) | 96.0 | (10.7) | .197 |
| Alcohol consumption g/day, md (IQR) | 6.3 | (1.8−15.3) | 11.0 | (3.8−27.1) | 9.0 | (2.9−22.8) | 6.5 | (1.2−18.3) | <.001 |
| High risk drinker, n (%) | 18 | (5) | 39 | (14) | 37 | (10) | 34 | (12) | .001 |
| Current smoker, n (%) | 50 | (14) | 62 | (22) | 95 | (25) | 64 | (23) | .002 |
| Married or cohabiting, n (%) | 300 | (84) | 233 | (83) | 301 | (79) | 195 | (69) | <.001 |
| High education, n (%) | 102 | (29) | 82 | (29) | 82 | (22) | 45 | (16) | <.001 |
| Employed, n (%) | 326 | (92) | 256 | (91) | 334 | (90) | 229 | (84) | .014 |
| Perceived health, n (%) |  |  |  |  |  |  |  |  | <.001 |
| Fair, poor or very poor | 104 | (29) | 79 | (28) | 134 | (35) | 128 | (46) |  |
| Good or very good | 258 | (71) | 204 | (72) | 244 | (65) | 153 | (54) |  |
| HSCL-25 at age of 31 years |  |  |  |  |  |  |  |  |  |
| Major symptoms, score >1.75, n (%) | 15 | (4) | 12 | (4) | 24 | (6) | 35 | (12) | <.001 |
| Female |  |  |  |  |  |  |  |  |  |
| Age, years, mean (SD) | 46.6 | (0.6) | 46.5 | (0.6) | 46.5 | (0.6) | 46.7 | (0.6) | .001 |
| Height, (cm), mean (SD) | 164.6 | (6.1) | 165.0 | (5.9) | 164.9 | (5.7) | 164.1 | (6.3) | .241 |
| Weight, (kg), mean (SD) | 71.4 | (14.0) | 72.0 | (14.3) | 72.6 | (16.3) | 71.6 | (15.5) | .634 |
| BMI, (kg/m2), mean (SD) | 26.3 | (4.9) | 26.4 | (5.0) | 26.7 | (5.8) | 26.6 | (5.5) | .773 |
| WC, (cm), mean (SD) | 86.6 | (12.4) | 86.7 | (12.4) | 87.6 | (13.9) | 87.2 | (13.8) | .729 |
| Alcohol consumption g/day md, (IQR) | 2.2 | (0.4−6.3) | 3.8 | (1.0−9.4) | 3.0 | (0.8−8.2) | 2.6 | (0.5−8.2) | .001 |
| High risk drinker, n (%) | 26 | (6) | 30 | (7) | 41 | (9) | 27 | (7) | .392 |
| Current smoker, n (%) | 70 | (16) | 90 | (22) | 87 | (19) | 58 | (16) | .084 |
| Married or cohabiting, n (%) | 350 | (80) | 320 | (78) | 367 | (80) | 273 | (75) | .254 |
| High education, n (%) | 124 | (29) | 143 | (36) | 126 | (28) | 75 | (21) | <.001 |
| Employed, n (%) | 385 | (90) | 368 | (91) | 391 | (88) | 291 | (83) | .002 |
| Perceived health, n (%) |  |  |  |  |  |  |  |  | <.001 |
| Fair, poor or very poor | 113 | (26) | 113 | (28) | 157 | (34) | 154 | (42) |  |
| Good or very good | 322 | (74) | 297 | (72) | 299 | (66) | 212 | (58) |  |
| HSCL-25 at age of 31 years |  |  |  |  |  |  |  |  |  |
| Major symptoms, score >1.75, n (%) | 19 | (4) | 32 | (8) | 52 | (11) | 59 | (16) | <.001 |

Note. p-value for Chi-Square test, MD – Median, IQR – Interquartile range, SD – Standard deviation, WC – Waist circumference, BMI – Body mass index, TCI – Cloninger’s temperament and Character Inventory, persistent – high in persistence, overactive – high in novelty seeking and low in harm avoidance, dependent – highest attachment and dependence, passive – high in harm voidance and low in novelty seeking. HSCL-25 – The Hopkins symptom checklist, severity of anxiety and depression symptoms
