# Supplementary material for: Temperament and longitudinal changes in physical activity – the Northern Finland Birth Cohort 1966 Study
Source: BMC Public Health. 2023 Mar 3;23:426. doi: 10.1186/s12889-023-15303-9 (PMC9985204; doi:10.1186/s12889-023-15303-9)
Supplement: Supplementary file 5 [file 12889_2023_15303_MOESM4_ESM.odt]

Additional file 4 

The mean temperament trait scores with standard deviations across physical activity groups according to change in leisure-time moderate 

to vigorous physical activity level from the age of 31 to the age of 46 years.

|  | Stable low (ref.) | Increased | Decreased | Stable high | p-value* |
| --- | --- | --- | --- | --- | --- |
| Variable |  |  |  |  |  |
| Temperament traits at the age of 31 |  |  |  |  |  |
| Male, n (%) | 737 (57) | 218 (17) | 180 (14) | 160 (12) |  |
| Novelty seeking | 19.3 (5.8) | 19.3 (5.8) | 20.6 (6.0)* | 19.4 (6.1) | .064 |
| Harm avoidance | 13.4 (6.1) | 12.6 (5.7) | 11.7 (5.6)** | 11.9 (6.2)** | < .001 |
| Reward dependency | 13.0 (3.7) | 13.7 (3.6)* | 13.0 (3.8) | 13.6 (3.7) | .021 |
| Persistence | 4.4 (1.8) | 4.7 (1.7) | 4.6 (1.8) | 5.0 (1.6)* | .002 |
| Female, n (%) | 1003 (60) | 325 (19) | 177 (11) | 163 (10) |  |
| Novelty seeking | 20.7 (6.1) | 20.4 (5.8) | 21.5 (5.9) | 21.1 (5.7) | .190 |
| Harm avoidance | 15.8 (6.0) | 14.8 (5.7)* | 14.4 (5.8)* | 12.8 (5.6)*** | < .001 |
| Reward dependency | 16.1 (3.4) | 16.1 (3.5) | 15.9 (3.6) | 16.3 (3.0) | .805 |
| Persistence | 3.9 (1.7) | 4.3 (1.6)** | 4.3 (1.7)* | 4.5 (1.8)** | < .001 |

Note. *Overall p-value refers to comparison between PA groups with the use of independent-samples analysis of variance (ANOVA), Post Hoc Dunnet t-test treat stable low group as a control, and compare all other groups against it, *p < .05, **p < .01, ***p < .001, MVPA – moderate to vigorous physical activity, Stable low – low MVPA level at the ages of both 31 and 46 years; Increased – low MVPA level at 31 but high at age 46; Decreased – high MVPA level at 31 but low at age 46; Stable high – high MVPA level at the ages of both 31 and 46 years.
